# Preparation of Core-Shell Hybrid Materials by Producing a Protein Corona Around Magnetic Nanoparticles

**DOI:** 10.1186/s11671-015-0992-2

**Published:** 2015-07-08

**Authors:** A Weidner, C Gräfe, M von der Lühe, H Remmer, J H Clement, D Eberbeck, F Ludwig, R Müller, F H Schacher, S Dutz

**Affiliations:** Institute of Biomedical Engineering and Informatics (BMTI), Technische Universität Ilmenau, Gustav-Kirchhoff-Straße 2, 98693 Ilmenau, Germany; Klinik für Innere Medizin II, Abteilung Hämatologie und Internistische Onkologie, Universitätsklinikum Jena, Erlanger Allee 101, 07747 Jena, Germany; Institute of Organic and Macromolecular Chemistry, Friedrich-Schiller-University Jena, Humboldtstraße 10, 07743 Jena, Germany; Jena Center for Soft Matter (JCSM), Friedrich-Schiller-University Jena, Philosophenweg 7, 07743 Jena, Germany; Institut für Elektrische Messtechnik und Grundlagen der Elektrotechnik, Technische Universität Braunschweig, Hans-Sommer-Straße 66, 38106 Braunschweig, Germany; Physikalisch-Technische Bundesanstalt, Abbestraße 2-12, 10587 Berlin, Germany; Department of Nano Biophotonics, Leibniz Institute of Photonic Technology (IPHT), A.-Einstein-Straße 9, 07745 Jena, Germany

**Keywords:** Magnetic nanoparticles, Protein corona, Zeta potential, Hybrid materials, Core-shell particles, 87, 87.14.E-, 87.85.Qr

## Abstract

Nanoparticles experience increasing interest for a variety of medical and pharmaceutical applications. When exposing nanomaterials, e.g., magnetic iron oxide nanoparticles (MNP), to human blood, a protein corona consisting of various components is formed immediately. The composition of the corona as well as its amount bound to the particle surface is dependent on different factors, e.g., particle size and surface charge. The actual composition of the formed protein corona might be of major importance for cellular uptake of magnetic nanoparticles. The aim of the present study was to analyze the formation of the protein corona during in vitro serum incubation in dependency of incubation time and temperature. For this, MNP with different shells were incubated in fetal calf serum (FCS, serving as protein source) within a water bath for a defined time and at a defined temperature. Before and after incubation the particles were characterized by a variety of methods. It was found that immediately (seconds) after contact of MNP and FCS, a protein corona is formed on the surface of MNP. This formation led to an increase of particle size and a slight agglomeration of the particles, which was relatively constant during the first minutes of incubation. A longer incubation (from hours to days) resulted in a stronger agglomeration of the FCS incubated MNP. Quantitative analysis (gel electrophoresis) of serum-incubated particles revealed a relatively constant amount of bound proteins during the first minutes of serum incubation. After a longer incubation (>20 min), a considerably higher amount of surface proteins was determined for incubation temperatures below 40 °C. For incubation temperatures above 50 °C, the influence of time was less significant which might be attributed to denaturation of proteins during incubation. Overall, analysis of the molecular weight distribution of proteins found in the corona revealed a clear influence of incubation time and temperature on corona composition.

## Background

Magnetic nanoparticles (MNP) represent perfectly suitable materials for a variety of biomedical and biotechnological applications. In many cases, MNP have to penetrate into different cell types of living tissue. This tissue and cellular uptake is strongly influenced by the particle size, as well as its surface chemistry and modification with functional groups or biomolecules. A detailed investigation and clarification of the interactions between surface chemistry of the particles and living tissue is a key to understand and control cellular uptake mechanisms [1].

Upon application of nanoparticles into biological media (e.g., whole-blood or plasma), the formation of a protein “corona” around the particles takes place immediately. This corona is a completely closed protein monolayer of a few nanometers on the surface of the nanoparticles [2] and can be divided into a “soft” and a “hard” corona [3]. In the *soft corona*, a permanent exchange of macromolecules from the surrounding medium and the particle surface takes place and this leads to a fast and persistent variation of the structure of the *soft corona*. The *hard corona* consists of macromolecules rather fixed to the particle surface and shows a more or less temporal constant composition. The volatility of the proteins in the *soft corona* aggravates a detailed investigation of the influence of the unstable part of the corona and thus most work has been spent on the investigation of the *hard corona* [4]. It was found that the corona is much more complex than previously considered. The influence of different particle parameters on corona formation has been already investigated. Lundquist and co-workers [5] found that for a fixed type of material, the biologically active proteins in the corona are strongly determined by the size as well as the zeta potential of the particles. Furthermore, it is well-known that the adsorption of blood serum proteins to particles is time-dependent [6]. Proteins with the highest mobility are bound to the surface first and later they will be replaced by less motile proteins which show a higher affinity to the surface. This process can take several hours. Casals et al. [7] confirmed that a *soft* corona loosely attached to the particles surface changes to an irreversibly attached *hard* corona over time. Comprehensive review articles about the influence of different nanoparticle parameters (e.g., composition, size, shape, crystallinity, surface area, surface defects, charge, roughness, transfer capability, and hydrophobicity/hydrophilicity) on the corona composition were published in the past years by several authors [4, 8–13].

Other less investigated but very important factors influencing the corona composition are the temperature at which the incubation of particles and protein source takes place as well as the duration of the incubation. For the clarification of this issue, the major aim of this study was the investigation of the influence of incubation temperature and time on the composition of the corona. For this, magnetic iron oxide nanoparticles with different coatings and, thus, different zeta potential were incubated with fetal calf serum (FCS) at different temperatures and incubation times. Magnetic nanoparticles enable an effective magnetic washing and separation which is very advantageous for the handling of small amounts of sample. In this study, the incubation was carried out at a homogeneous temperature in the whole sample in a water bath. The formed corona and the agglomeration behavior of the incubated particles were investigated as a function of incubating temperature and time by different methods.

## Methods

### Preparation of MNP

The superparamagnetic iron oxide nanoparticles used in this paper were prepared similar to the well-known wet chemical precipitation method [14] but using another alkaline medium [15]. For this, a 1.17-M NaHCO_3_ solution was directly added to a FeCl_2_/FeCl_3_ solution with a Fe^2+^/Fe^3+^ ratio of 1:1.7, and a brownish precipitate occurred. After the addition of distilled water, the particles were boiled for 5 min at 100 °C. In this way, single-core MNP were formed under the release of CO_2_, and the color of the solution turned black. Afterwards, the obtained MNP suspension was washed twice by magnetic separation with distilled water using a high-performance permanent magnet to remove excess educts.

For the investigation of the influence of particle surface charge on the formation of the protein corona, MNP were coated with different materials (dextran (DEX), carboxymethyl-dextran (CMD), and diethylaminoethyl-dextran (DEAE)). These materials have a neutral dextran backbone but different substitution patterns and thus enable a variation of surface charge (DEX—neutral, CMD—negative, DEAE—positive). For coating the MNP with dextran and its derivatives, the nanoparticles were dispersed by ultra-sonic treatment (Sonopuls GM200, BANDELIN electronic, Berlin, Germany) for 30 s. HCl was added to adjust the pH value at 2 to 3, and the suspension was tempered at 45 °C in a water bath and stirred. At the same time, the coating agents were dissolved in distilled water in a mass-ratio (coating/core) of 1:1. The so-prepared coating solution was steadily added to the nanoparticle suspension and stirred for 1 h at 45 °C. Afterwards, the suspension was treated with ultrasound for 30 s again, washed magnetically two times with distilled water to remove coating material excess, and the desired concentration was adjusted by adding distilled water.

Due to limited stability against agglomeration of dextran-based coatings, MNP with a coating of poly(*tert*-butoxycarbonyl acrylic acid) (P*t*BAA) were used for some investigations [16]. This particle system shows high stability against agglomeration and thus allows the minimization of the influence of agglomeration on corona formation. For coating of MNP with PtBAA, 40 mg of PtBAA were dissolved in 40 mL MilliQ water at pH = 12. To this solution, 40 mL of a dispersion of MNP (1 g/L) was added. The mixture was stirred at 50 °C for 1 h, the dispersion was centrifuged with 8000 rpm for 30 min, and the supernatant was removed. The particles were redispersed in MilliQ water using ultrasonication. This procedure was repeated five times.

All prepared nanoparticle suspensions show a stability against sedimentation of several months as described in previous investigations [17, 18].

### Serum Incubation of the MNP

For producing a protein corona around MNP, the particles have to be incubated in a natural protein source which leads to an accumulation of proteins on the surface of the MNP. For our studies, FCS was used as natural protein source. FCS incubation of MNP was performed by water bath heating resulting in a homogeneous temperature distribution throughout the sample. In the following sections, uncoated and coated magnetic nanoparticles are referred as MNP and serum-incubated MNP are referred as MNP@Corona.

For water bath incubation, FCS was tempered at defined temperatures (incubation temperature) in a water bath. Fifteen milligrams of coated MNP from previously prepared suspensions were filled up with 2 ml of tempered FCS and kept at the same temperature (incubation temperature) for a certain time (incubation time). Incubation time starts with the application of FCS. During the time of incubation, ultra-sonic treatment at a given temperature was carried out (S100H, Elmasonic, Germany) to re-disperse possible agglomerates. At defined incubation time points (1, 5, 10, and 20 min), the suspensions were taken out of the water bath and put on a magnet for magnetic separation, excess FCS was withdrawn and distilled water was added.

The washed incubated nanoparticle suspensions were kept at 4 °C for short-term storage or at −80 °C for long-time storage.

### Thermogravimetric Analysis

A suitable way to determine the mass of the corona bound to the particle surface is thermogravimetric analysis (TGA). Therefore, uncoated MNP were incubated (25 °C/10 min in FCS), and resulting fluids were freeze dried to obtain fine dry powders for TGA experiments. These samples were heated (STA409, Netzsch, Selb, Germany) from room temperature up to 330 °C, and the corresponding mass loss was continuously determined. The obtained curves for MNP@Corona were normalized to curves for uncoated MNP.

### Structural and Magnetic Characterization

Magnetic core size was determined by X-ray diffraction (XRD, X’Pert PRO, PANalytical, The Netherlands) and using the Scherrer formula as well as by transmission electron microscopy (TEM; 200 kV FEI Tecnai G^2^ 20, equipped with a 4k × 4k Eagle HS CCD and a 1k × 1k Olympus MegaView camera for overview images).

The magnetic properties were measured by vibrating sample magnetometry (VSM; Micromag TM 3900, Princeton Measurement Systems, USA). Measurement was performed on liquid samples or dried powders. The concentration of MNP within the liquid samples and the amount of proteins bound to particle surface were calculated from the obtained saturation magnetization. The overall magnetic behavior of the samples was derived from coercivity and relative remanence.

### Magnetorelaxometry

Magnetorelaxometry (MRX) was performed to investigate the Brownian relaxation behavior [19] of the MNP and the MNP@Corona hybrids. The relaxation curves describe the decay of an initial magnetization (after a magnetization pulse from a coil) due to Brownian and Néel relaxation of the particles within a fluid. From these relaxation curves, the size and size distribution of the particles was calculated by fitting the so-called cluster moment superposition model (CMSM) to the relaxation data [20]. The distribution of the hydrodynamic diameters *d*_h_ or cluster diameters *d*_c_ is assumed to be a lognormal one. Previous investigations showed a good agreement with hydrodynamic diameters obtained by dynamic light scattering (DLS) [21].

In the study presented here, we applied two different setups for MRX. A setup measuring the magnetic relaxation by means of highly sensitive low-T_c_-SQUID sensors at a distance of 10 mm above the sample (SQUID-MRX) [22] was used to investigate the agglomeration behavior of different coated MNP and resulting MNP@Corona in detail. For the measurement of the kinetics of corona formation around MNP, a setup which utilizes fluxgate sensors (FG-MRX) for measuring the magnetization decay was used [23].

In SQUID-MRX, samples were magnetized for 1 s with a magnetic field of 2 mT and relaxation was measured in a time window of 450 μs to 0.5 s after magnetization pulse. For FG-MRX investigations, magnetic moments of MNP were aligned in a field of 2 mT for 2 s duration and relaxation of the sample net magnetic moment was measured over a time period of 1.5 s after the magnetization pulse.

Since superparamagnetic nanoparticles show no thermally blocked magnetism, they relax predominantly via Néel relaxation. To observe a Brownian relaxation in MRX, larger ferrimagnetic nanoparticles of about 50 nm [17, 24] were used here in the presented MRX studies. Of course, this results in higher absolute particle and agglomerate sizes than for superparamagnetic cores, but we suppose that the overall behavior of corona formation and agglomeration, investigated on ferrimagnetic cores, is similar to that of superparamagnetic cores.

### Measurement of the Surface Charge

To determine the surface charge of the MNP and MNP@Corona hybrids, the zeta potential is a valid and widely used parameter. For this measurement, a Zetasizer (Nano ZS, Malvern, UK) and appropriate software (Zetasizer ver. 6.20) were used. Before the measurement, samples were diluted in the ratio 1:30 with distilled water and treated in an ultrasonic bath. The medium viscosity and dielectric constant were taken from water at 25 °C with 0.8 cP and 0.8872, respectively. Measurements were performed in three consecutive runs and obtained values were averaged.

### Gel Electrophoresis

The determination of the composition of the protein corona on the surface of MNP@Corona hybrids was carried out by means of sodium dodecyl sulfate-polyacrylamide gel electrophoresis (SDS-PAGE). For this, 2 × Laemmli sample buffer (Bio-Rad, Munich, Germany) supplemented with 2-mercaptoethanol (final concentration 355 mM) was added to the samples in the first step and heated up to 95 °C to crack secondary and tertiary structure of proteins. Then, the denatured proteins were separated by molecular weight with PAGE on a 4–12 % Bis-Tris gel (Bio-Rad, Munich, Germany). After the run, the proteins were visualized by highly sensitive silver staining (SilverXpress Silver Staining Kit (Invitrogen, Heidelberg)). Gel images were processed by ImageJ (National Institutes of Health, Bethesda, USA) [25].

As references, a molecular weight standard protein collection Kaleidoscope marker (Bio-Rad, Munich, Germany) and untreated FCS were used.

## Results and Discussion

**Fig. 1:**
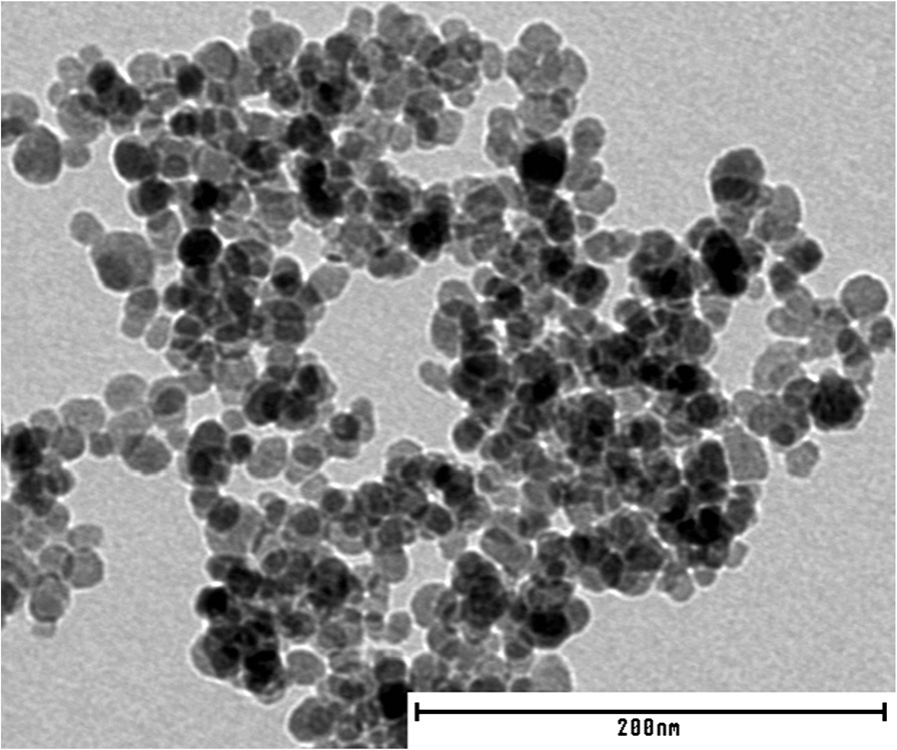
Typical TEM image of as-prepared magnetic nanoparticles. Particle agglomeration occurs during preparation (drying) of colloidal stable fluids for TEM investigation

The core diameter of MNP was determined by means of XRD and TEM to be around 10 nm (Fig. 1). Analysis of the diffractogram confirmed a spinel structure of the prepared particles with maghemite (γ-Fe_2_O_3_) as dominant magnetic phase.

**Fig. 2:**
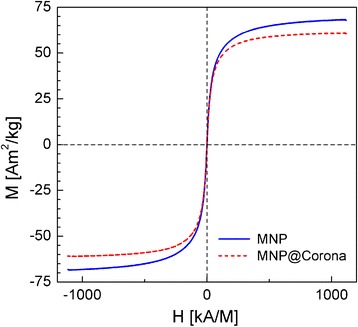
Hysteresis curves (VSM) of uncoated MNP (*blue solid line*) and protein corona-coated MNP (MNP@Corona; *red dashed line*) confirm superparamagnetic behavior of the particles as well as a solid fraction of non-magnetic proteins of about 10 % by mass for dried protein corona-coated samples

These data are confirmed by measurements of the static magnetization-versus-magnetic-field curves (Fig. 2). The saturation magnetization (*M*_S_) of freeze-dried samples of uncoated MNP is 68.2 Am^2^/kg, which is a typical value for maghemite. Resulting from a coercivity (*H*_C_) of less than 0.2 kA/m and a relative remanence (*M*_R_/*M*_S_) of about 0.005 at room temperature, the used particles show superparamagnetic behavior. Estimation of magnetic core size following the Chantrell method [26] provides a mean core size of 9.6 nm. This value is in good accordance with results from XRD and TEM [18].

For the investigation of the amount of proteins bound to the particle surface, uncoated MNP were incubated in FCS for 10 min at 25 °C and obtained MNP@Corona were freeze dried to a powder after magnetic washing of the sample to remove the excess FCS. This MNP@Corona powder shows an *M*_s_ of 60.8 Am^2^/kg. Taking into account a nonmagnetic behavior of the protein coating, this decrease in saturation magnetization corresponds to a fraction of about 10 % by mass of proteins included in the hybrid particles. Although within this study we cannot prove whether serum incubation of MNP leads to a homogeneous layer of proteins on the particle surface or rather to protein bundles containing some MNP, we use the term “coated” for the serum incubated particles throughout the manuscript.

**Fig. 3:**
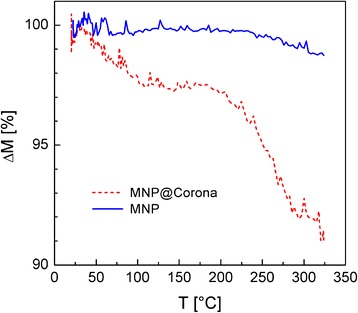
TGA curves of uncoated MNP (*blue solid line*) and MNP@Corona (*red dashed line*) confirm a solid fraction of non-magnetic proteins of 8 % by mass for dried samples after incubation

This composition of the freeze-dried MNP@Corona was confirmed by TGA measurements (Fig. 3). For temperatures up to 330 °C, TGA measurements of the native particles show a weight loss of about 1 % which can be attributed to the evaporation of adsorbed water and a possible phase transformation of a small amount of impurity phases (hydrated oxides and hydroxides of iron) to hematite. Compared to this, TGA investigation of protein corona-coated MNP provides a mass loss of about 9 % in two steps. Again, the first step corresponds to the evaporation of adsorbed water and impurity phase change to hematite, whereas in the second step, the surface proteins decompose. Normalizing this curve to the losses in pure particles, a corona mass of 8 % is obtained. This value is slightly below the value from VSM. Although this difference probably is within the error of both TGA and VSM, another reason might be incomplete decomposition/evaporation of proteins during heating up to 330 °C.

**Fig. 4:**
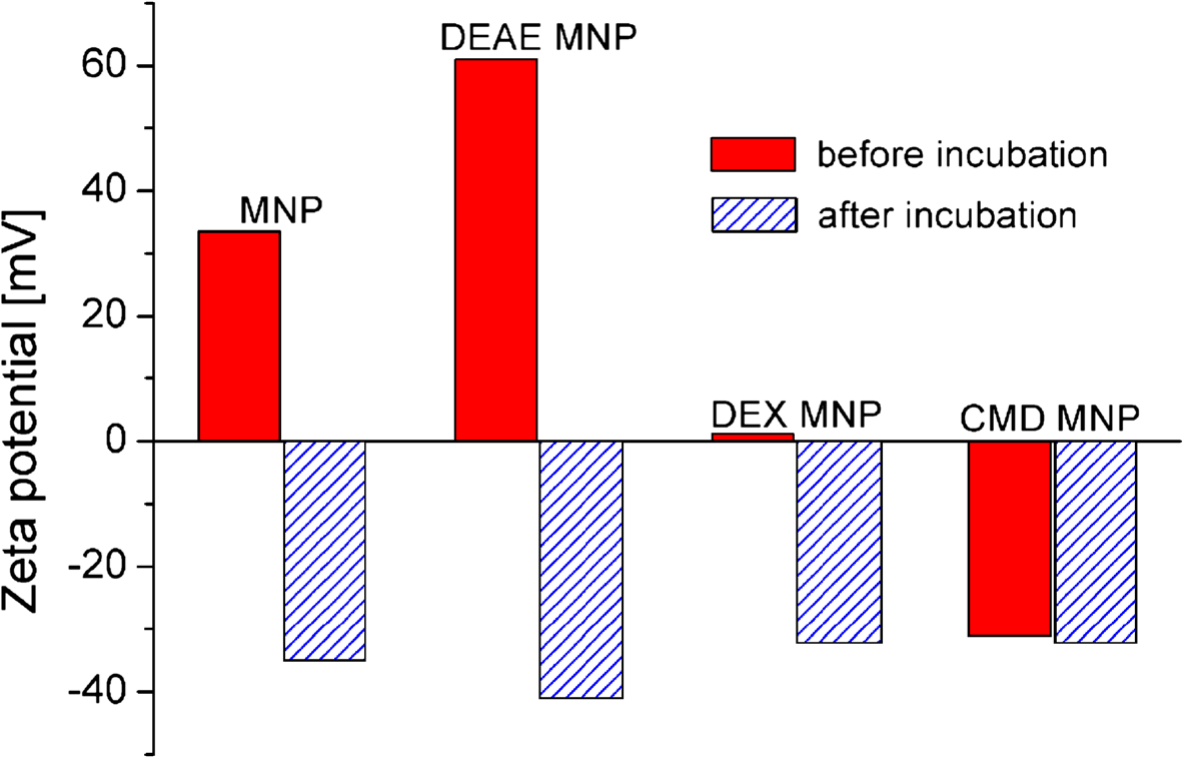
Zeta potential of MNP and for MNP coated with DEAE-dextran, dextran, and CM-dextran before (*red columns*) and after serum incubation (*blue hatched columns*) confirms formation of a protein corona around magnetic nanoparticles during serum incubation

The results regarding the occurrence of a nonmagnetic layer on the particle surface after FCS incubation of MNP confirm the formation of a protein corona around MNP. A further evidence for a successful protein corona formation is given by changes in surface charge of particles after serum incubation. Figure 4 shows the zeta potential of pure MNP and for MNP coated with DEAE-dextran, dextran, and CM-dextran before and after serum incubation.

It is clearly demonstrated that serum incubation significantly changes the surface charge of particles. Independent of the surface charge of particles before incubation, serum-treated particles reveal a negatively charged surface showing a zeta potential in the range from −32 to −41 mV. Since proteins and their subunits as well as other serum components (e.g., lipids) have a negative charge at pH 7, this fact can be interpreted as a confirmation of the formation of a protein corona around magnetic nanoparticles. Furthermore, a certain influence of the initial surface charge on the resulting zeta potential after incubation can be seen. This behavior has been already described by [5] and has to be investigated in more detail in further studies.

In measurements after different storage times, it was found that the formed protein corona is stable for about 9 days at room temperature and then starts to decay. To prevent the wash off of the protein corona during storage, serum-incubated samples are stored in a slight excess FCS.

Cell toxicity investigations (CellTiter-Glo and PrestoBlue) revealed no toxic effect of bare cores, coated cores, as well as pure coating materials (DEX, CMD, DEAE, and P*t*BAA) on tested cell lines (human brain microvascular endothelial cells) [18]. Another major factor for the suitability of nanoparticles for in vivo applications is their agglomeration behavior. Agglomerates larger than a few micrometers bear the risk to occlude blood vessels (especially capillaries) which may lead to an embolization and thus serious side effects for patients. In most cases, agglomeration cannot be prevented completely and thus it has to be tolerated as long as agglomerates are well below 1 μm [27]. To exclude any risk from agglomerates, the agglomeration behavior of the samples has to be investigated.

Applying dynamic light scattering (DLS) methods, agglomeration behavior of particle suspensions can be assessed by detecting an increase in hydrodynamic diameter. However, the interpretation of the polydispersity index (PDI) in terms of the width of the size distribution is challenging, in particular because of the strong weighting of larger objects (∝*V*^2^, *V*—volume of the scattering objects). Because of its valuable information for the interpretation of aggregation, the size distribution of aggregates or clusters was evaluated by SQUID magnetorelaxometry (SQUID-MRX). The size distribution is assumed to be of lognormal form and is expressed in the diameter of mean cluster volume, *d*_vc_, i.e., the mean volume equivalent cluster diameter, the mean of volume weighted size distribution, *d*_wvc_, and the geometric dispersion parameter *σ*_c_ (Table 1), derived from the CMSM fit. It was shown earlier that *σ*_c_ correlates well with visually observable precipitation and that the “z average” diameter of DLS ranges between *d*_vc_ and *d*_wvc_, depending on *σ*_c_ [20].

**Table 1:** Parameters of the distribution of volume equivalent hydrodynamic diameters of MNP before and after incubation in FCS obtained by fitting of CMSM to MRX data. Alternatively, *σ*_c_ was fixed with fitting indicated by “(fix),” in order to render the mean diameters comparable

Sample	*d*_vc_ (nm)	*d*_wvc_ (nm)	*σ*_c_
Dextran	125	227	0.55
Dextran@Corona	155	261	0.51
Dextran@Corona	141	257	0.55 (fix)
DEAE	118	231	0.58
DEAE@Corona	60	216	0.80
DEAE@Corona	137	268	0.58 (fix)
CMD	106	207	0.58
CMD@Corona	82	177	0.62
CMD@Corona	96	188	0.58 (fix)

In the following, we quantitatively discuss the changes in the size distribution caused by dispersion in FCS compared to the original aqueous MNP dispersion. Incubation of neutrally charged dextran-coated MNP with FCS reduces *σ*_c_ slightly and increases the mean diameter *d*_vc_ by 16 nm (while *σ*_c_ was fixed at the reference value, Table 1). This behavior might be attributed to the growth of an additional layer of 8 nm thickness onto the MNP shell.

In case of the transfer of positively charged DEAE-coated MNP into FCS, the dispersion parameter *σ*_c_ grows dramatically. Such a behavior might refer to agglutination as a possible mechanism of aggregation [28]. Accordingly, the comparison of mean diameters is hard to interpret. After fixing *σ*_c_ at the reference value while fitting the CMSM to the data of MNP in FCS, again an increase of *d*_vc_ was found, here by 19 nm. However, if MNP really agglutinate, it cannot be derived from the present data whether the MNP got homogeneously covered by an opsonisation layer or not.

Also in case of a negatively charged CMD shell, the dispersion parameter increases during exposure to FCS. In contrast to the DEAE sample, the mean diameters, obtained while fixing *σ*_c_, decrease (Table 1). Again, significant broadening of the size distribution points to the aggregation or transformation of existing aggregates. Thus, quantitative answers about opsonisation cannot be made.

**Fig. 5:**
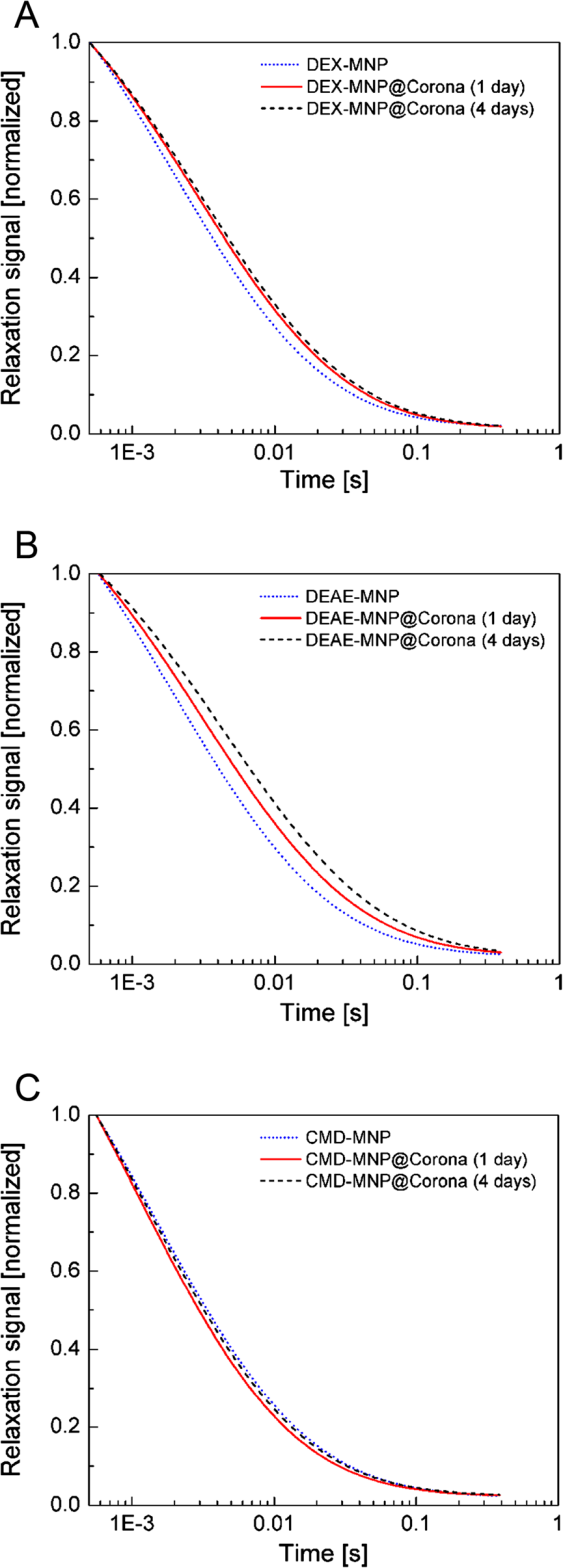
MRX curves for magnetic nanoparticles coated with dextran **(a)**, DEAE-dextran **(b)**, and CMD-dextran **(c)** before (*blue dotted lines*) and 1 day (*red solid lines*) as well as 4 days (*black dashed lines*) after incubation. The curves were normalized with respect to the undetermined background and amplitude

To get an additional impression of agglomeration behavior, relaxation curves were qualitatively analyzed (Fig. 5). In case of neutrally charged dextran-coated MNP (Fig. 5a), serum incubation of these particles leads to a slight increase of relaxation time in comparison to the undiluted original sample which probably is caused by an increasing particle size due to the growth of a protein corona on the particle surface. Also, a slight agglomeration might explain the observed effect, as discussed later for DEAE-coated MNP. However, the decrease of *σ*_c_ (Table 1) is a clear indicator for an increase of the particle diameter, and we regard it as rather unlikely to originate from aggregation. Aging of the samples for 4 days leads to a minor increase in relaxation time which might be caused most probably by further growth of the opsonisation layer or possibly by cross-linking between surface proteins [29, 30].

MNP with DEAE-dextran (Fig. 5b) showed similar tendencies like dextran-coated particles but stronger variances in relaxation behavior of investigated samples. Serum incubation of DEAE-dextran-coated MNP led to distinctly higher relaxation times and, thus, a larger amount of protein corona on particle surface can be supposed. This behavior is even more pronounced after aging for 4 days, possibly due to further growth of corona or due to protein cross-linking. A possible explanation might be that there is a stronger affinity between negatively charged proteins and highly positively charged surface of DEAE-dextran-coated particles than for particles with pure dextran or CM-dextran coating. From this, a higher protein load on the surface may be caused which results in a larger effective particles size and thus a higher relaxation time. This hypothesis has to be verified in further studies by means of measurements, providing data for the amount of proteins bound to particles.

For CMD-coated MNP, also significant changes in relaxation behavior for different samples were found (Fig. 5c). In contrast to dextran and DEAE-dextran for CMD-coated MNP, the relaxation gets faster after FCS incubation. However, as mentioned above, the increase of *σ*_c_ indicates some aggregation or even disaggregation of already present aggregates. Note that the obtained *d*_v_ around 100 nm is significantly larger than the core diameter of 10 nm (Fig. 1). After aging the sample for 4 days, the difference in relaxation behavior vanishes and a relaxation curve similar to that before serum incubation is found, possibly also due to a further growth of a corona or due to cross-linking between particles. By means of MRX, a clear change of the cluster size distribution due to the FCS incubation was shown. But it cannot be distinguished whether an opsonisation or an aggregation is responsible for the observations. So one may speculate that no protein corona is formed during serum incubation of these samples since there was no significant change in zeta potential (Fig. 4) observed, and CMD as well as most proteins in FCS is negatively charged. At least partially, opsonisation is supported by experimental evidence of surface proteins by means of gel electrophoresis investigations for FCS-incubated CMD-coated MNP as shown in own previous investigations [31].

**Fig. 6:**
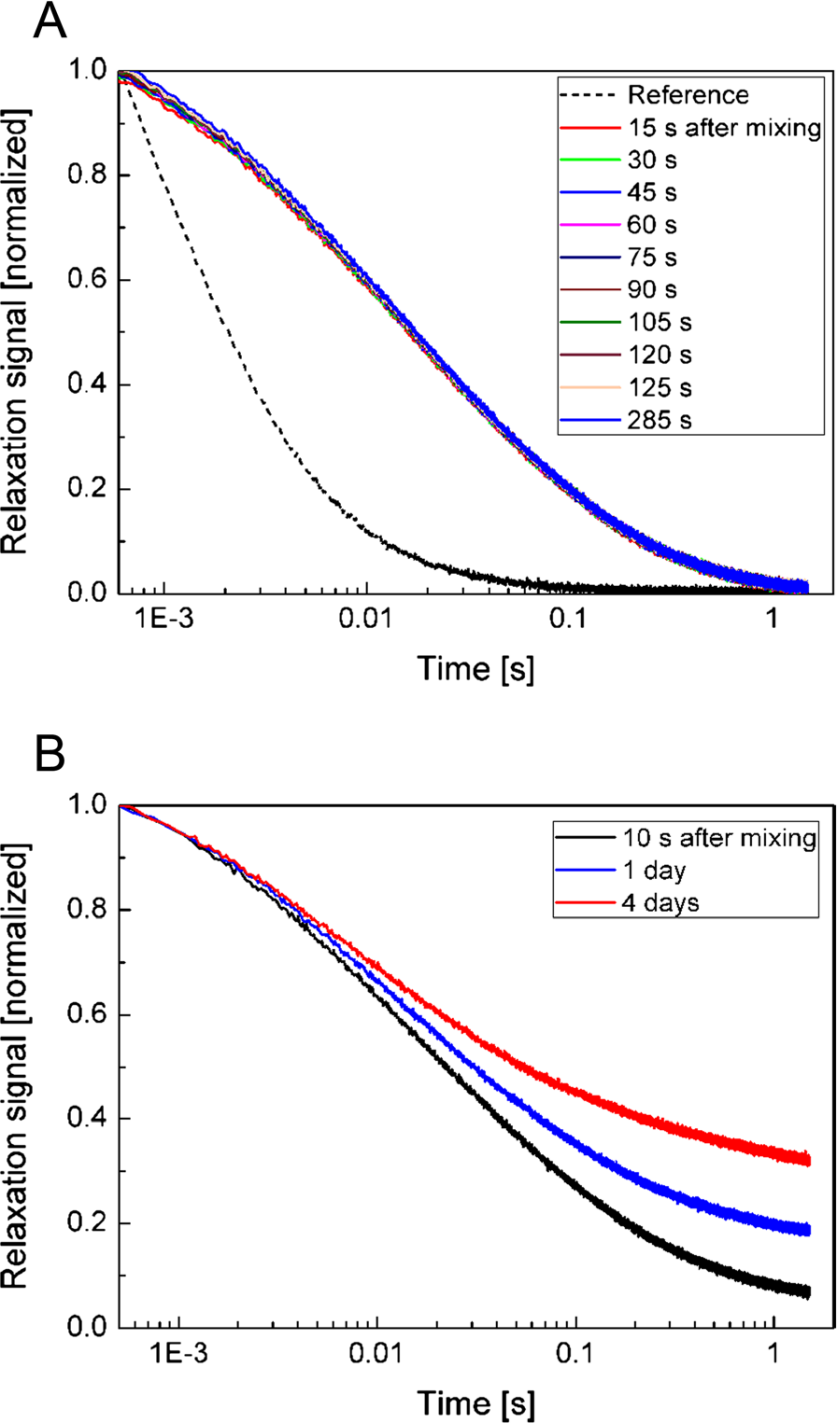
Temporal evolution of the MRX signal after adding 140 μL FCS to 10 μL DEAE-coated MNP suspension for the first 285 s after mixing **(a)** and for incubation times up to 4 days **(b)**, curves are normalized to be “1” for time point 1 ms. For comparison, MRX signal measured on reference sample (10 μL DEAE-coated MNP suspension diluted with 140 μL DI H_2_O) is shown in **(a)**

Since corona formation on DEAE-coated MNP leads to a significant effect on relaxation time, this MNP type was used to investigate the protein corona formation dynamics by means of fluxgate MRX. For this, MRX measurements were performed immediately after adding FCS to a MNP suspension at room temperature. Figure 6a depicts the temporal evolution of the MRX signal. The first unaveraged measurement was recorded 15 s after adding FCS to the ferrofluid, and then measurements were repeated every 15 s. As can be seen, no changes are discernable. The decay of magnetization after the addition of FCS is slower compared to the aqueous suspension (same volume H_2_O was added to MNP suspension). This increase of the relaxation time constant is mainly caused by an increase of the effective hydrodynamic size by agglutination [28] since the viscosity of FCS is with 1.56 mPa·s only about 50 % higher than that of water.

From the relaxation curves, it can be seen that the formation of a protein corona occurs immediately within seconds, and no clearly visible changes are detected over the observed time period (up to 285 s). This conclusion is valid only for the thickness of the corona and does not reflect any changes in corona composition. However, on a longer time scale (Fig. 6b), a further change of relaxation time was observed for 1 and 4 days of incubation. There are two possible reasons for such a behavior. First, a further growth of the protein corona over a long time might occur, which is relatively unlikely. The more probable reason for the continuous decrease of relaxation time might be attributed to cross-linking of surface proteins as discussed above which results in larger agglomerates [29, 30].

Altogether, it can be stated from MRX measurements that the formation of a protein corona occurs immediately after mixing MNP and FCS and that the resulting corona has (depending on the underlying coating material) an effect on particle agglomeration in aqueous MNP@corona hybrid particle suspensions. Agglomerates with sizes below 250 nm in diameter, as observed here, can be tolerated for medical application of investigated particles.

**Fig. 7:**
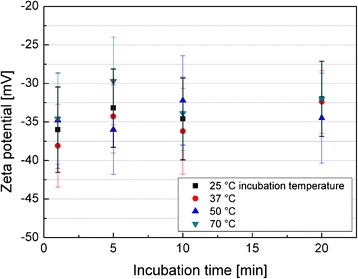
Zeta potential of serum-incubated P*t*BAA-coated MNP as function of incubation time and temperature

In order to study the impact of incubation time and temperature in more detail, we used a MNP system with high stability against agglomeration and sedimentation to exclude any influence of particle agglomeration on the corona formation. For this, we used particles coated with poly(*tert*-butoxycarbonyl acrylic acid) (P*t*BAA), a negatively charged polyelectrolyte [16]. P*t*BAA-coated MNP show a negative surface charge, similar to earlier observations after coating MNP with CMD. These MNP were incubated with FCS for up to 20 min within a temperature range from 25 to 70 °C (Fig. 7). The different temperatures were realized by heating in a water bath.

Zeta potential measurements of P*t*BAA-MNP@Corona as function of incubation time and temperature reveal that the formation of the protein corona does not alter the overall net charge of these materials (Fig. 7). Serum proteins are negatively charged at pH 7.2 in fetal calf serum. Therefore, the overall negative charge of the nanoparticles remains constant between −40 and −30 mV in all cases.

Nevertheless, when trying to derive a model for the influence of incubation time and temperature on the composition of the resulting corona, the zeta potential is not a suitable measure to determine details (since no clear correlations between incubation parameters and resulting surface charge can be found) but rather a raw indicator for changes in the structure of the protein corona.

**Fig. 8:**
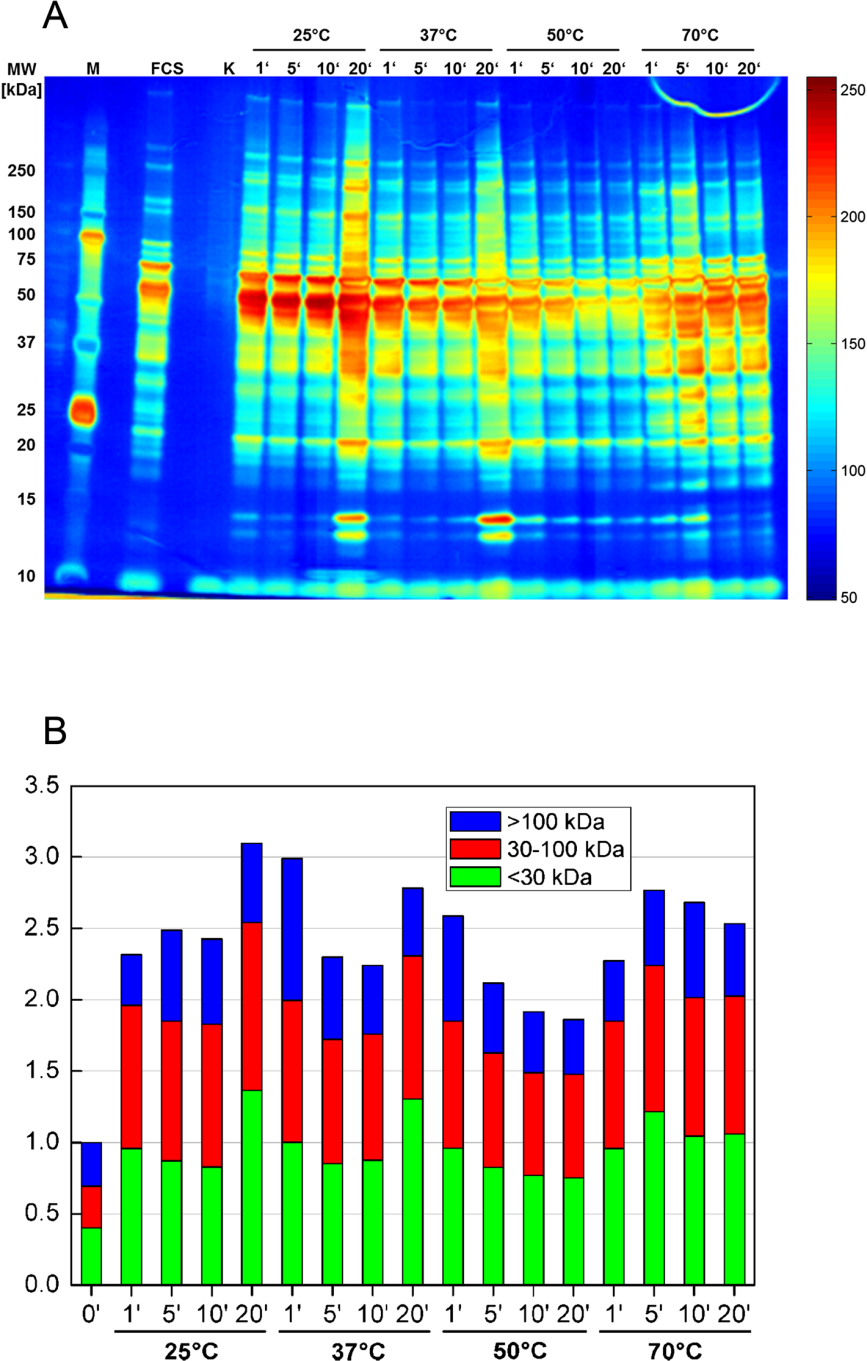
Pseudocolor image of SDS-PAGE gel (4–12 % Bis-Tris) of P*t*BAA-coated magnetic nanoparticles after serum incubation for different incubation times and temperatures (*K* = untreated MNP “0”) **(a)** and quantitative analysis of the same SDS-PAGE gel of bound protein amount and raw estimation of protein’s molecular weight distribution **(b)**

To get more profound information about the protein load and composition of the formed protein corona, P*t*BAA samples were subjected to SDS-PAGE. With this approach, components of the corona were denatured and separated according to their molecular weight (Fig. 8a).

At 25 and 37 °C, an increase of protein content with time is visible, whereas at 50 °C, a more or less steady distribution is observed. Please note that the overall protein distribution reflects the situation in untreated FCS. Figure 8b shows a quantitative analysis of the protein distribution. It can be clearly seen that only weak differences in the bound protein amount exist within the first 10 min of incubation. This result is confirmed by MRX where all samples show more or less identical relaxation behavior within the first 5 min. However, for heating times of 20 min, a significant increase in bound protein mass occurs. Since this effect was found for 25 and 37 °C incubation series as well as in MRX investigations (1-day curve), we exclude an experimental artifact.

At 70 °C, a higher protein yield is detected independent of the incubation time. Almost all of the serum proteins are denatured above 65 °C and thus misfolded polypeptides and protein agglomerates are formed during incubation and attach to the surface of PtBAA-coated MNP. Some of these clusters could not be resolved by conventional lysis conditions and cause an accumulation of polypeptides in the range between 25 and 75 kDa. The loss of high-molecular weight proteins might be due to the temperature-related structural changes, too. Unspecific intramolecular bounds lead to more globular shapes which exhibit a higher electrophoretic mobility. This observation needs more detailed analysis of the distinct proteins which are involved in corona formation.

Additionally, Fig. 8b provides an impression on the composition of protein corona. It becomes obvious that heating time and temperature have an influence on corona composition. Since SDS-PAGE analysis and zeta potential investigations provide a global overview on corona composition, other methods have to be utilized for a detailed clarification of protein corona composition on polypeptide level. A promising method for this task is matrix-assisted laser desorption/ionization (MALDI) in combination with a mass spectrometer which is tested in ongoing studies.

## Conclusions

Our MRX investigations show that immediately after contact of MNP with a protein source (FCS), a protein corona is formed on the particle surface. This leads to an increase of particles size and, depending on any previously applied MNP coating, to a slight agglomeration of the MNP during the first minutes of incubation. Longer incubation (from hours to days) leads to stronger agglomeration of corona-coated MNP, probably due to cross-linking of the surface proteins. We quantified the amount of proteins bound to MNP under these conditions by a combination of magnetic measurements and thermogravimetry to about 10 %.

Independent of the used polymer shells herein used for MNP (DEAE, CMD, dextran, P*t*BAA), zeta potentials from −30 to −40 mV were found after serum incubation. Slight variations in the zeta potential of the serum-incubated MNP are a first hint towards differences in composition of the formed protein corona at different incubation times and temperatures, possibly as also the coating material play a role. Quantitative SDS-PAGE analysis of serum-incubated particles revealed, as already found by MRX, a relatively constant amount of bound proteins during the first minutes of serum incubation. After longer incubation (20 min), a considerably higher amount of surface proteins was determined for incubation temperatures of 25 and 37 °C. For incubation temperatures of 50 and 70 °C, the incubation time did not seem to play a major role, which might be attributed to denaturation of proteins during incubation.

The analysis of the molecular weight of proteins found in the corona showed a clear influence of incubation time and temperature on corona composition which has to be investigated in more detail in future studies by means of MALDI. Furthermore, magnetic nanoparticles can be used in prospective investigation for magnetic heating by means of reversal losses in an alternating magnetic field [32, 33] during the incubation to control the composition of the corona by using a temperature gradient from the particle surface to the surrounding protein source.

## Abbreviations

CMD: carboxymethyl-dextran
DEAE: diethylaminoethyl-dextran
DEX: dextran
DLS: dynamic light scattering
FCS: fetal calf serum
MNP: magnetic nanoparticles
MRX: magnetorelaxometry
PDI: polydispersity index
P*t*BAA: poly(tert-butoxycarbonyl acrylic acid)
TEM: transmission electron microscope
TGA: thermogravimetric analysis
VSM: vibrating sample magnetometry
XRD: X-ray diffraction
